# Serum Trimethylamine-*N*-Oxide and Its Precursors as a Diagnostic Biomarker Panel for Non-Muscle-Invasive Bladder Cancer

**DOI:** 10.3390/ijms27083591

**Published:** 2026-04-17

**Authors:** Aleyna Baltacıoğlu, Osman Acar, Ceyda Sönmez, Yeşim Sağlıcan, Ömer Burak Argun, Ali Rıza Kural, Asıf Yıldırım, Ümit İnce, Muhittin Abdulkadir Serdar, Aysel Özpınar

**Affiliations:** 1 Department of Biochemistry and Molecular Biology, Graduate School of Health Sciences, Acibadem Mehmet Ali Aydinlar University, Istanbul 34752, Turkey; aleyna.baltacioglu@live.acibadem.edu.tr (A.B.); ceyda.sonmez@live.acibadem.edu.tr (C.S.); muhittin.serdar@acibadem.edu.tr (M.A.S.); 2 Department of Medical Biochemistry, School of Medicine, Acibadem Mehmet Ali Aydinlar University, Istanbul 34752, Turkey; osman.acar@live.acibadem.edu.tr; 3 Department of Pathology, School of Medicine, Acibadem Mehmet Ali Aydinlar University, Istanbul 34752, Turkey; yesim.saglican@acibadem.com (Y.S.); umit.ince@acibadem.com (Ü.İ.); 4 Medical Laboratory Techniques, Health Services of Vocational School, Istanbul Kent University, Istanbul 34413, Turkey; drburakargun@gmail.com; 5 Department of Urology, School of Medicine, Acibadem Mehmet Ali Aydinlar University, Istanbul 34752, Turkey; ali.kural@acibadem.com; 6 Department of Urology, School of Medicine, Istanbul Medeniyet University, Istanbul 34722, Turkey; asifyildirim@yahoo.com

**Keywords:** non-muscle-invasive bladder cancer, trimethylamine-*N*-oxide (TMAO), carnitine, choline, betaine, LC–MS/MS, metabolomics, biomarker discovery, ROC analysis, cancer metabolism

## Abstract

Non-muscle-invasive bladder cancer (NMIBC) is characterized by high recurrence rates and necessitates lifelong cystoscopic surveillance, underscoring the need for minimally invasive biomarkers to improve early detection and risk stratification. Therefore, this study aimed to investigate the role of trimethylamine-*N*-oxide (TMAO) and its precursors as diagnostic biomarkers for NMIBC. A total of 50 male patients with NMIBC (25 pTa and 25 pT1) were included in this study. Additionally, 52 age-matched healthy individuals were included as controls. Serum TMAO and its dietary precursors were quantified using liquid chromatography–tandem mass spectrometry. Group differences were analyzed using nonparametric tests, associations were assessed using Spearman’s correlation, and diagnostic performance was evaluated using receiver operating characteristic (ROC) analysis. Multivariate logistic regression was performed to identify independent predictors, and a composite risk score was generated. Serum TMAO, carnitine, and choline levels were significantly higher in patients with NMIBC than in controls (*p* ≤ 0.0001), whereas betaine showed a nonsignificant trend toward higher levels (*p* ≥ 0.05). The pathological stage (pTa vs. pT1) showed the strongest correlation with TMAO levels. The ROC analysis revealed that TMAO had the highest individual diagnostic accuracy (area under the curve [AUC] = 0.875, 95% confidence interval [CI] 0.812–0.939), whereas carnitine and choline provided complementary diagnostic performance. In multivariate models, TMAO, carnitine, and choline remained independent predictors of NMIBC (*p* ≤ 0.0001). A composite risk score integrating all four metabolites demonstrated excellent discriminatory capacity (AUC = 0.958, 95% CI 0.926–0.991). The TMAO metabolic axis can be used as a minimally invasive biomarker panel for NMIBC. Further large, prospective, multicenter studies integrating metabolomic and microbiome profiling are needed to validate the findings.

## 1. Introduction

Bladder cancer is one of the most prevalent malignancies of the urinary tract worldwide [1]. It is the ninth most commonly diagnosed cancer globally (614,298 new cases) and the 13th leading cause of cancer-related death, accounting for 220,596 deaths worldwide (GLOBOCAN 2022). Additionally, the number of new cases is expected to exceed 650,000 annually in the coming years, reflecting a steadily increasing global burden. This underscores the urgent need for early, accurate, and minimally invasive diagnostic strategies.

Bladder cancer exhibits a marked sex disparity, with a higher incidence in men, whereas women are frequently diagnosed at more advanced stages and exhibit poorer outcomes [2]. In 2022, the age-standardized incidence rates were approximately 27–28 per 100,000 men in Southern Europe and were substantially lower in women, highlighting a pronounced global sex disparity (GLOBOCAN 2022). Clinically, bladder cancer includes a heterogeneous spectrum of neoplasms. Non-muscle-invasive bladder cancer (NMIBC; pTa and pT1) is associated with favorable survival but high recurrence rates. Therefore, long-term surveillance is necessary. Conversely, muscle-invasive disease (≥pT2) is characterized by aggressive behavior, early metastatic spread, and substantially higher mortality [3]. Histologically, urothelial carcinoma accounts for approximately 90% of cases, with squamous cell carcinoma and adenocarcinoma representing less common variants [4].

The etiology of bladder cancer involves a complex interplay between environmental exposure, genetic alterations, and epigenetic deregulation [5]. Cigarette smoking is the strongest risk factor for developing bladder cancer, driven by the urinary excretion of carcinogenic aromatic amines [6]. Occupational exposure to similar compounds in dye, textile, and petrochemical industries further increases the risk [7]. Furthermore, chronic inflammatory conditions, including recurrent infections, long-term catheterization, and infection with *Schistosoma haematobium*, are associated with squamous differentiation [8]. At the molecular level, tumor initiation and progression are driven by frequent alterations, including FGFR3, TP53, and TERT promoter mutations, along with widespread genomic instability and aberrant DNA methylation [9].

**Figure 1:**
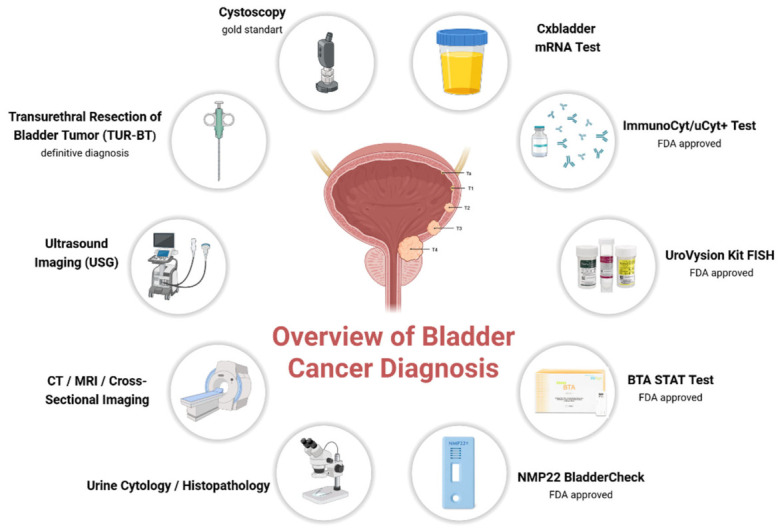
Overview of current diagnostic methods for bladder cancer. Abbreviations: TUR-BT, transurethral resection of bladder tumor; CT, computed tomography; MRI, magnetic resonance imaging; USG, ultrasonography; NMP 22, nuclear matrix protein 22; BTA, bladder tumor antigen; FISH, fluorescence in situ hybridization. Created in BioRender. Baltacıoğlu, A. (2026) https://BioRender.com/gfibcqr (accessed on 26 February 2026).

Despite advances in clinical management, early diagnosis remains a clinical challenge [10]. Cystoscopy is the gold standard for diagnosing bladder cancer (Figure 1). However, it is invasive, costly, and burdensome for patients [11]. Urine cytology has high specificity for high-grade tumors but lacks sensitivity for low-grade tumors [12]. Food and Drug Administration-approved urinary biomarker assays, including NMP22, BTA, UroVysion fluorescence in situ hybridization (FISH), and ImmunoCyt/uCyt, have improved detection but remain limited by variable performance and difficulties in standardization (Figure 1) [13]. These limitations underscore the need for minimally invasive, reliable, and reproducible biomarkers [14]. Serum-based metabolite profiling has emerged as a promising strategy, offering greater stability and resistance to confounding factors, such as hydration status, variable urinary pH, and dilution effects inherent in urine samples [15].

**Figure 2:**
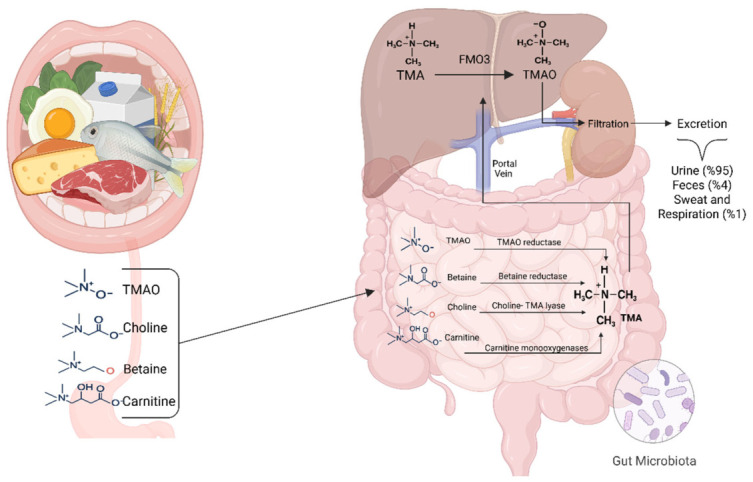
Overview of TMAO biosynthesis and excretion. Dietary precursors choline, L-carnitine, betaine, and TMAO are absorbed in the intestine and metabolized by the gut microbiota into trimethylamine (TMA). TMA enters the portal circulation and is oxidized to TMAO by hepatic flavin-containing monooxygenase 3 (FMO3). Circulating TMAO is predominantly eliminated via renal filtration into urine, with minor excretion in feces, sweat, and exhaled air. Adapted from “Trimethylamine-*N*-Oxide (TMAO) as a Rising-Star Metabolite: Implications for Human Health” (Metabolites 2025, 15, 220) [26], licensed under CC BY 4.0. Created in BioRender. Baltacıoğlu, A. (2026) https://BioRender.com/x6lfisy (accessed on 26 February 2026).

With recent advances in metabolomics, the opportunities for biomarker discovery in oncology have expanded [16]. Metabolomics offers a direct representation of the tumor phenotype and its interaction with the host by capturing systemic metabolic alterations [17]. The role of host–microbiota interactions and their metabolites in cancer biology is of particular interest [18]. Trimethylamine-*N*-oxide (TMAO), derived from dietary precursors such as choline, carnitine, and betaine via gut microbial metabolism and hepatic oxidation by FMO3, has been implicated in multiple oncogenic processes [19]. Choline, carnitine, and betaine are essential precursors for TMAO biosynthesis and play independent roles in tumor metabolism, including membrane synthesis and mitochondrial energy flux. Therefore, their collective analysis is crucial for obtaining a comprehensive metabolic profile. Experimental studies have shown that TMAO promotes inflammation through NF-κB activation, induces oxidative and endoplasmic reticulum stress, and stimulates angiogenesis via VEGF signaling [20]. Because TMAO is predominantly excreted in urine, the bladder urothelium is chronically exposed to this metabolite, which raises the possibility of a localized carcinogenic effect [21]. Although associations between TMAO and several cancers, including colorectal cancer, have been reported, its role in bladder cancer remains largely unexplored [22]. TMAO levels can be influenced by dietary habits and gut microbiota composition [23]. Mediterranean diet and other dietary interventions have been reported to modulate circulating TMAO levels; these findings highlight the diet-dependent nature of this metabolite [24]. Population-based studies in Turkey indicate that adherence to Mediterranean diet-type regimens varies across regions and demographic groups, reflecting notable dietary heterogeneity [25]. Such variability may, in turn, contribute to interindividual differences in baseline TMAO levels, particularly in populations with diverse dietary patterns. Figure 2 shows an overview of TMAO biosynthesis from dietary precursors, including gut microbiota-dependent trimethylamine formation, hepatic conversion, and renal excretion.

This study aimed to evaluate TMAO and its precursors collectively in the context of bladder cancer. For this purpose, we quantitatively analyzed serum levels of TMAO and its dietary precursors choline, carnitine, and betaine in patients with NMIBC compared with healthy controls using liquid chromatography–tandem mass spectrometry (LC–MS/MS) [27]. We hypothesized that these metabolites could be significantly altered in bladder cancer and provide diagnostic value as noninvasive biomarkers [28]. This study addresses an important gap in the field and provides insights into novel metabolic markers for early detection and risk stratification [29].

## 2. Results

### 2.1. Cohort Characteristics and Baseline Metabolite Levels

**Figure 3:**
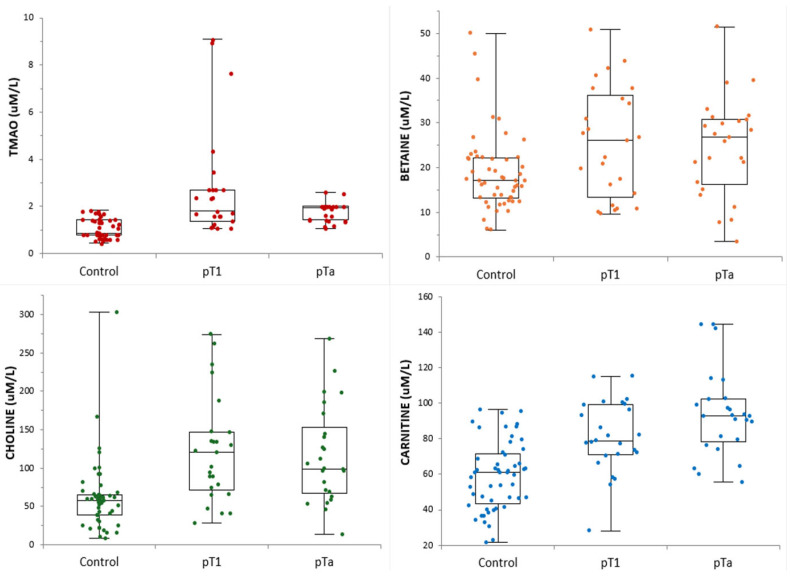
Serum concentrations of TMAO and its precursors in each clinical group. Box-and-whisker plots with individual data points show serum levels of TMAO, betaine, carnitine, and choline in healthy control participants, patients with pT1, and patients with pTa. Abbreviations: TMAO, trimethylamine-*N*-oxide; pTa, noninvasive papillary urothelial carcinoma; pT1, lamina propria-invasive urothelial carcinoma; µM/L, micromoles per liter.

Table 1 shows the baseline demographic and clinical characteristics and serum metabolite levels of the study population. No significant differences in age, body mass index (BMI), height, and weight were observed between the three groups (*p* > 0.05). Figure 3 shows the distribution of serum metabolite levels across the groups.

### 2.2. Serum Metabolite Alterations in Patients with Bladder Cancer

Comparisons showed marked differences in serum metabolite profiles between healthy controls and patients with NMIBC (Table 1). This indicates that the observed metabolic alterations are independent of basic clinical characteristics.

The Kruskal–Wallis test showed strong overall group effects for TMAO, carnitine, and choline (*p* < 0.0001), whereas betaine showed only a weak, nonsignificant trend toward higher levels in the tumor groups (*p* = 0.094). The post hoc Bonferroni-adjusted Mann–Whitney *U* test showed that serum carnitine, choline, and TMAO levels were significantly higher in the pTa and pT1 groups than in the controls (*p* < 0.05), consistent with the clear separation observed in the box plots (Figure 3). Conversely, betaine levels did not differ significantly between the controls and either tumor group, despite numerically higher median levels in the pTa group.

No significant differences in metabolite levels were observed between the pTa and pT1 groups, indicating a broadly similar metabolic phenotype across non-muscle-invasive stages within this cohort. Therefore, subsequent analyses focused on the most informative comparison: healthy controls versus NMIBC (pTa + pT1 combined).

### 2.3. Correlation Between Serum Metabolites, Tumor Stage, and Anthropometric Variables

**Figure 4:**
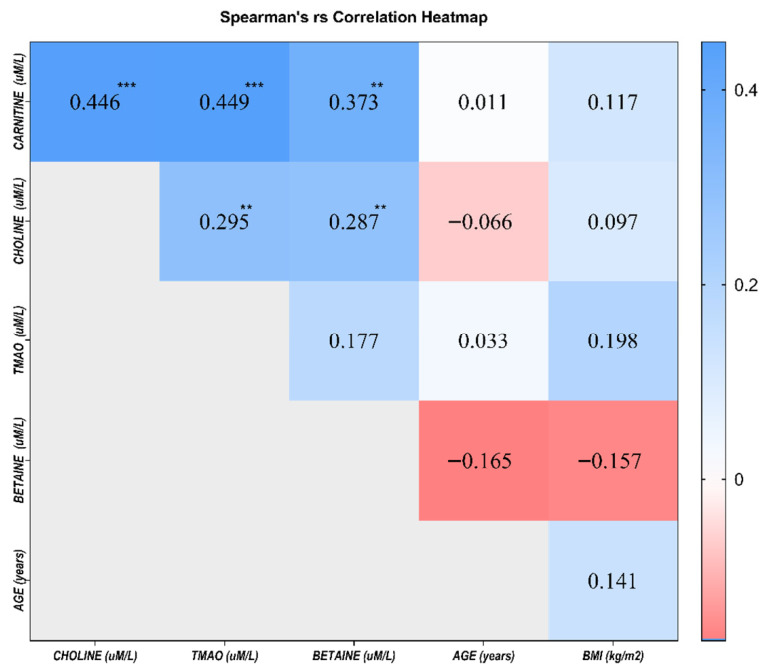
Spearman’s rank correlation heatmap of serum TMAO, its precursors, and clinical variables. Numbers represent rs values, whereas asterisks represent the level of statistical significance (** *p* < 0.01, and *** *p* < 0.001; two-tailed). Blue shades indicate positive correlations, red shades indicate negative correlations, and white indicates no or minimal correlation.

Spearman’s rank correlation analysis was performed to investigate the relationships between serum TMAO, its precursors, and clinical variables (Figure 4 and Table S1). Carnitine, choline, and TMAO showed moderate positive intercorrelations: carnitine was correlated with choline and TMAO (rs = 0.446 and rs = 0.449, respectively; *p* < 0.001), and choline was correlated with TMAO (rs = 0.295, *p* < 0.01). Betaine was weakly correlated with the other metabolites, showing modest positive correlations with carnitine and choline (rs = 0.373 and rs = 0.287, respectively; *p* < 0.01), whereas its correlation with TMAO was very weak and nonsignificant (rs = 0.177).

Age and BMI showed weak and nonsignificant correlations with the metabolites (rs ≤ 0.20, *p* > 0.05). Age was not correlated with any of the four biomarkers, and BMI showed a very weak, nonsignificant positive correlation with TMAO (rs = 0.198). These findings indicate that the observed associations between tumor stage and metabolite levels are only minimally influenced by age or BMI.

### 2.4. Diagnostic Performance of Individual Metabolites, Multivariate Model, and Composite Risk Score

**Figure 5:**
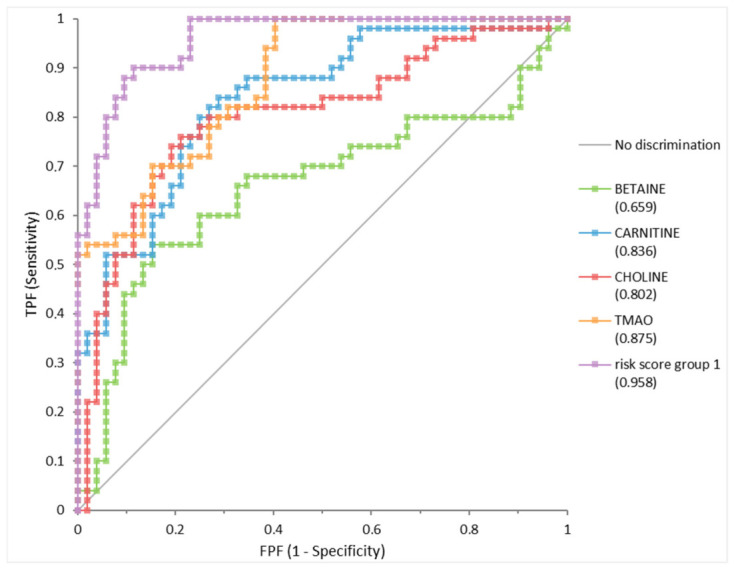
ROC curves for individual serum metabolites and the composite risk score for differentiating NMIBC from healthy controls. Abbreviations: AUC, area under the curve; CI, confidence interval; TMAO, trimethylamine-*N*-oxide; ROC, receiver operating characteristic.

The receiver operating characteristic (ROC) curve analysis confirmed the strong discriminative performance of TMAO, carnitine, and choline in distinguishing NMIBC from healthy controls (Figure 5). Among the individual metabolites, TMAO showed the highest diagnostic accuracy (area under the curve [AUC] = 0.875, 95% confidence interval [CI] 0.812–0.939). A cutoff value of 1.48 μM/L yielded moderate sensitivity (72.3%) and high specificity (84.6%), indicating that TMAO has a stronger ability to correctly identify nondiseased individuals (low false-positive rate) than to detect all true positive cases.

Carnitine (AUC = 0.836, 95% CI 0.759–0.913) and choline (AUC = 0.802, 95% CI 0.714–0.891) demonstrated more balanced diagnostic profiles, with sensitivities of 84% and 76% and specificities of 71.2% and 78.8%, respectively. Conversely, betaine displayed limited diagnostic utility (AUC = 0.659, 95% CI 0.548–0.771) (Table 2). Cutoff values for all metabolites were derived from the maximum Youden index, and binary classifications based on these cutoffs showed statistically significant intergroup differences in Fisher’s exact test (*p* < 0.0001), although the performance for betaine was clearly weaker.

All four metabolites were included in a multivariate logistic regression model to examine their joint contribution (Table S2). The analysis confirmed the independent predictive value of carnitine, choline, and TMAO for bladder cancer detection. TMAO showed the strongest association with NMIBC (β = 4.115, odds ratio [OR] = 61.23, 95% CI 7.201–520.7, *p* < 0.0002), whereas carnitine (β = 0.07141, OR = 1.074, 95% CI 1.027–1.123, *p* = 0.001) and choline (β = 0.02485, OR = 1.025, 95% CI 1.008–1.043, *p* = 0.004) showed moderate but significant associations. Betaine did not reach statistical significance (β = −0.014, OR = 0.986, 95% CI 0.915–1.062, *p* = 0.712). The overall model was statistically robust (likelihood-ratio test: χ^2^(4) = 88.18, *p* < 0.0001).

A composite risk score was derived based on the regression coefficients using the following model equation:

$$\begin{array}{cc} \mathrm{logit}\;(\mathrm{bladder}\;\mathrm{cancer}) & \\ = & -13.04\;(intercept)\text{–}0.014\times \mathrm{Betaine}\\ + & 0.07141\times \mathrm{Carnitine}+0.02485\times \mathrm{Choline}\\ + & 4.115\times \mathrm{TMAO}. \end{array}$$

When assessed using ROC analysis alongside the individual metabolites, this composite risk score markedly improved diagnostic accuracy. The risk score achieved an AUC of 0.958 (95% CI 0.926–0.991), demonstrating excellent discriminative power and clearly outperforming any single metabolite (Figure 5). An optimal cutoff value of 12.86 yielded high sensitivity (90%) and specificity (89%), with a Youden index of 0.785. Fisher’s exact test confirmed highly significant discrimination between patients with early-stage bladder cancer and controls (*p* < 0.0001), and the corresponding OR indicated a markedly increased risk of bladder cancer above the cutoff (OR = 53.813, 95% CI 15.167–239.482). Table 2 shows the summary metrics of this composite risk score alongside those for the individual metabolites.

Considering the modest sample size and study design, this multivariate model and the derived risk score should be considered exploratory. We deliberately focused on serum metabolite concentrations as predictors because age and BMI were broadly comparable between groups and showed weak, nonsignificant correlations with the metabolites. Therefore, the reported OR and AUC estimates are likely to be overestimated and require external validation in larger, independent cohorts.

The diagnostic metrics of the identified serum metabolite panel were compared with those reported for commonly used urine-based tests for bladder cancer detection to place its performance in a clinical context (Table 3). Although the composite serum risk score demonstrated high sensitivity (90%) and specificity (89%), currently available urine-based assays show variable performance across studies. Although no direct head-to-head comparison was performed, these findings indicate that systemic metabolite profiling may represent a complementary diagnostic strategy for NMIBC.

## 3. Discussion

This study investigated the serum concentrations of TMAO and its precursors choline, carnitine, and betaine in patients with NMIBC (stages pTa and pT1) compared with healthy controls. TMAO, carnitine, and choline levels were significantly higher in patients with NMIBC, whereas betaine exhibited limited discriminatory power. ROC analysis confirmed the diagnostic value of these metabolites, with TMAO demonstrating the strongest individual performance. Moreover, integrating TMAO, carnitine, and choline into a multivariate model yielded superior diagnostic accuracy (AUC = 0.96), highlighting the potential utility of a metabolite panel rather than relying on single biomarkers alone. This finding highlights the advantage of a panel-based approach over individual biomarkers in improving diagnostic performance. Furthermore, correlation analyses revealed positive associations between TMAO, carnitine, and choline and tumor stage, whereas age and BMI showed weak, nonsignificant correlations with these metabolites. These findings indicate that the observed metabolic alterations are more likely to reflect disease-related processes than to be driven primarily by host demographic or anthropometric factors.

These findings are consistent with accumulating evidence implicating TMAO and its precursors in tumor biology. TMAO is a gut microbiota-derived metabolite generated from dietary choline, betaine, and carnitine and has been reported to be associated with chronic inflammation, oxidative stress, and endothelial dysfunction [33,34]. Mechanistically, TMAO activates proinflammatory pathways, such as NF-κB; stimulates cytokine production, such as IL-6 and TNF-α; and promotes angiogenesis via VEGF upregulation [35]. Our observation that TMAO levels are markedly elevated in patients with NMIBC supports the hypothesis that TMAO may contribute to bladder carcinogenesis not only via systemic inflammatory and vascular effects but also through continuous exposure of the bladder mucosa during urinary excretion. Similarly, TMAO pretreatment increases the susceptibility of bladder epithelial cells to uropathogenic *Escherichia coli* infection, thereby amplifying chronic inflammation, an established risk factor for bladder cancer [36].

Choline and carnitine demonstrated significant diagnostic potential in our cohort. Both are essential precursors for TMAO biosynthesis. However, they may also contribute to tumor development via TMAO-independent mechanisms [37,38,39,40,41,42]. Choline plays a central role in membrane phospholipid synthesis and one-carbon metabolism. Dysregulation of choline metabolism has been reported to be associated with aberrant DNA methylation, altered epigenetic regulation, and oncogene activation [37,38,39]. Therefore, the elevated serum choline levels in our patients may reflect increased substrate availability for TMAO production and perturbations in methylation-related pathways that favor urothelial transformation. Carnitine is crucial for mitochondrial fatty acid transport and β-oxidation. Although the role of carnitine in cancer biology remains controversial, excessive carnitine-driven fatty acid metabolism has been reported to be associated with malignant metabolic reprogramming and tumor progression [40,41,42]. Our finding of higher carnitine levels in patients with NMIBC is compatible with this concept and with reports from patients with thyroid and other solid tumors. Conversely, betaine showed no significant discriminatory power. Betaine functions as an osmolyte and methyl donor and exerts protective and context-dependent protumorigenic effects. Although low betaine levels have been reported to be associated with impaired methylation capacity and genomic instability [43], the lack of a clear association in our cohort may reflect the complex and tissue-specific roles of betaine in tumor biology.

From a diagnostic perspective, these findings highlight several important considerations. TMAO alone demonstrated moderate sensitivity but relatively high specificity, suggesting a greater capacity to minimize false-positive classifications than to detect all true cases when used as a standalone biomarker. In contrast, choline and carnitine exhibited more balanced diagnostic profiles and showed markedly improved overall performance when integrated with TMAO into a composite risk score. In this study, the combined panel achieved an AUC of approximately 0.96 with high sensitivity and specificity, indicating strong potential for clinical application, particularly in risk stratification and clinical decision support rather than population-level screening. However, biomarker performance observed in exploratory case–control settings may be overestimated due to potential selection bias and study design constraints risk stratification; therefore, independent external validation is essential to confirm reproducibility. In addition, correlation analyses revealed that these metabolites were associated with tumor stage rather than age or BMI, supporting their robustness as disease-related markers in well-defined clinical settings. Although NMIBC represents a clinically important stage that is highly amenable to curative intervention, it remains challenging to detect using conventional diagnostic methods. Cystoscopy is invasive and costly, whereas urine cytology has limited sensitivity, particularly for low-grade disease. These limitations highlight the need for improved risk stratification and more accurate diagnostic approaches. Recent studies have further emphasize the importance of refined NMIBC classification systems to better guide clinical decision-making and patient management [44]. Therefore, minimally invasive blood-based biomarkers may complement current diagnostic algorithms.

The findings of this study contribute to the growing recognition of gut microbiota–cancer interactions. Because TMAO is generated from the microbial metabolism of dietary precursors, alterations in host–microbiota composition and function may represent an important axis in bladder carcinogenesis. Dysbiosis and increased microbial trimethylamine production lead to higher systemic TMAO levels, which in turn exacerbate chronic inflammation and oxidative stress [33,34]. The consistency between our metabolic findings and experimental evidence indicates that the TMAO axis warrants further investigation in bladder cancer.

The composite serum metabolite panel demonstrated comparable discriminative performance relative to currently available urine-based diagnostic tools (Table 3). Moreover, although urine-based assays reflect tumor cell shedding or genetic alterations, serum metabolites may capture systemic metabolic reprogramming and microbiota-related mechanisms. Considering that TMAO and its precursors are physiologically excreted in urine, a parallel urine-based metabolomic panel can further enhance noninvasive detection strategies in NMIBC.

This study has several limitations that should be acknowledged. First, only male individuals aged ≥50 years were included, which may have introduced selection bias; thus, the generalizability of the findings may be limited to female patients and younger populations. Second, the analysis was restricted to non-muscle-invasive tumors (pTa and pT1), as the study specifically focused on early-stage disease; therefore, the applicability of these findings to muscle-invasive bladder cancer remains unclear. Third, although LC–MS/MS offers high analytical sensitivity and specificity, the sample size was relatively small and the study design was case–control.

In addition, detailed dietary habits and smoking status were not systematically quantified, both of which may influence circulating metabolite levels and contribute to interindividual variability. Despite these potential confounders, the consistent and statistically significant differences observed in metabolite levels between the patients and control participants suggest that the identified metabolic alterations are more likely disease-associated rather than solely driven by dietary variability. Therefore, further studies with larger, independent cohorts and prospective longitudinal designs are needed to confirm the stability and reproducibility of these markers.

In addition to their diagnostic value, the findings highlight the translational potential of integrating metabolomics with existing diagnostic algorithms in bladder cancer. The superior performance of the composite metabolite panel indicates that serum-based profiling can complement and potentially reduce the need for invasive procedures in selected contexts. Future studies should prioritize validation in multicenter cohorts along with the integration of metabolomics, microbiota, and genomic and epigenomic data to elucidate mechanistic links and enable real-world implementation of metabolite-based signatures in personalized bladder cancer management.

## 4. Materials and Methods

**Figure 6:**
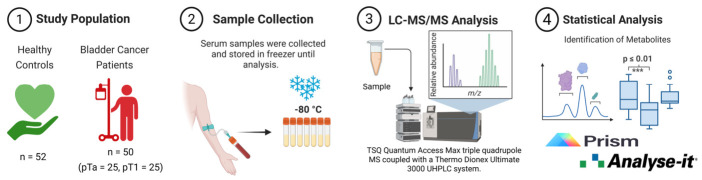
Study workflow. Abbreviations: LC–MS/MS, liquid chromatography–tandem mass spectrometry; UHPLC, ultra-high performance liquid chromatography; MS, mass spectrometer; pTa, pT1, pathological tumor stages a and 1. Created in BioRender. Baltacıoğlu, A. (2026) https://BioRender.com/zsd9txt (accessed on 14 April 2026).

This study was approved by the Clinical Research Ethics Committee of İstanbul Medeniyet University Göztepe Training and Research Hospital (GEAH) (2013-KAEK-64; decision no.: 2018/0367) and Ethics Committee of Acıbadem University (protocol code: ATADEK 2024-7/274) and conducted in accordance with the Declaration of Helsinki. Written informed consent was obtained from all participants. Figure 6 shows the study workflow.

### 4.1. Study Cohort

A total of 50 male patients aged ≥50 years with newly diagnosed primary NMIBC were included in this study. Additionally, 52 age-matched healthy individuals were included as controls. The NMIBC group comprised 25 patients with pTa and 25 with pT1 disease. Tumors were staged according to the 8th edition of the TNM Classification of Malignant Tumors and the European Association of Urology Guidelines on NMIBC [6,45]. In this classification, pTa indicates noninvasive papillary urothelial carcinoma confined to the urothelial mucosa, whereas pT1 refers to tumors invading the lamina propria without muscularis propria involvement. All patients were treatment-naïve at the time of blood collection.

**Figure 7:**
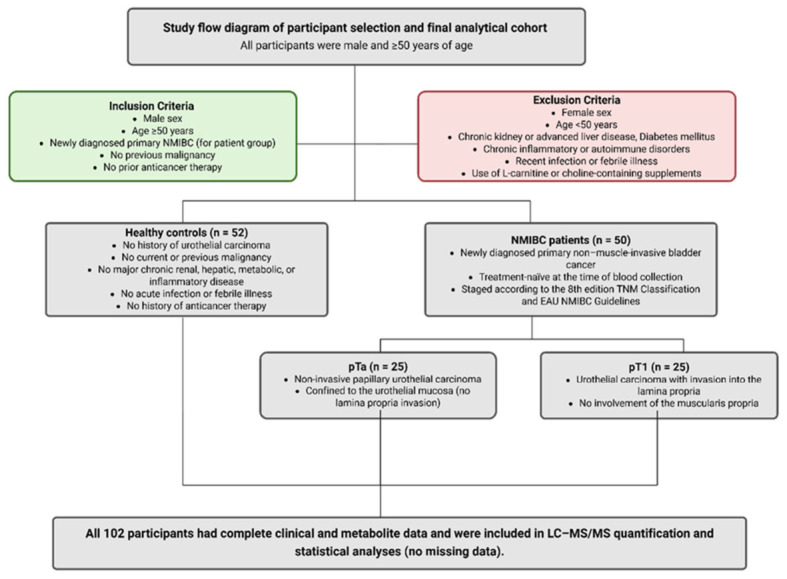
Study cohort flow diagram. Created in BioRender. Baltacıoğlu, A. (2026) https://BioRender.com/b2pf5qn (accessed on 26 February 2026).

The inclusion criteria were as follows: individuals with no previous diagnosis of bladder cancer or any other malignancy and no history of anticancer treatment, such as systemic chemotherapy, radiotherapy, or intravesical therapy. The exclusion criteria were as follows: individuals with major chronic diseases, such as chronic kidney disease, advanced liver disease, diabetes mellitus, inflammatory bowel disease, and autoimmune disorders; those with recent infection or febrile illness; and those with self-reported use of L-carnitine- or choline-containing dietary supplements or extreme dietary regimens (Figure 7).

### 4.2. Sample Collection

Serum samples were collected from healthy controls and patients with NMIBC under fasting conditions before transurethral resection of bladder tumor or any other oncological intervention. Samples were processed according to standard clinical procedures. Serum was divided into aliquots in polypropylene tubes and stored at −80 °C until LC–MS/MS analysis. All participants had complete serum metabolite and clinical data, and were included in the analysis.

### 4.3. Chemicals and Internal Standards

Analytical standards of TMAO (CAS No. 1184-78-7), choline (C7017), betaine (61962), and carnitine (8400920025) were obtained from Sigma-Aldrich (St. Louis, MO, USA). Isotopically labeled TMAO-d9 (Sigma-Aldrich) was used as the internal standard for TMAO quantification, whereas glipizide (Glucotrol XL^®,^ Pfizer Inc., New York City, NY, USA) was used as a general internal standard.

### 4.4. Sample Preparation and LC–MS/MS Analysis

Sample preparation and LC–MS/MS analysis of TMAO, choline, betaine, and carnitine were performed according to previously described protocols [41] with minor modifications. Briefly, serum metabolites were extracted by protein precipitation, and 5 μL of the supernatant was injected into the LC–MS/MS system. Quantitative analysis was performed using a Thermo Dionex Ultimate 3000 UHPLC system coupled with a TSQ Quantum Access Max triple quadrupole mass spectrometer (Thermo Scientific, San Jose, CA, USA). Multiple reaction monitoring transitions were optimized for each analyte, and calibration curves were constructed using authentic standards.

### 4.5. Statistical Analysis

Data analyses were performed using IBM SPSS Statistics v26.0 (IBM Corp., Armonk, NY, USA), Analyse-it v6.16.2 (Analyse-it Software, Ltd., Leeds, UK), and GraphPad Prism v9.0 (GraphPad Software, San Diego, CA, USA). Data normality was tested using the Shapiro–Wilk test. Normality testing (Shapiro–Wilk) and visual inspection indicated that several metabolites were not normally distributed, supporting the use of nonparametric methods in subsequent analyses. Therefore, continuous variables were presented as median (interquartile range). Between-group comparisons were performed using the Kruskal–Wallis test, followed by Bonferroni-adjusted Mann–Whitney *U* test. The diagnostic performance of metabolites was assessed using ROC curves, and the AUC, optimal cutoff, sensitivity, specificity, and Youden index were calculated. Multivariate logistic regression was used to evaluate the independent predictive value of serum metabolites and derive a composite risk score. A *p*-value ≤ 0.05 indicated statistical significance.

## 5. Conclusions

Serum TMAO, carnitine, and choline levels are significantly elevated in patients with NMIBC and exhibit promising diagnostic potential, particularly when evaluated as part of a combined metabolite panel. The findings provide valuable insights into the metabolic reprogramming associated with early-stage bladder cancer and support the concept that gut microbiota-derived and host-related metabolites along the TMAO axis may represent a biologically plausible link between microbial metabolism and urothelial tumorigenesis. Although the multivariate model and composite risk score showed excellent discriminatory performance, caution should be exercised in interpreting these results, and larger, multicenter studies are needed for independent external validation. Furthermore, the integration of serum metabolite profiling with microbiota characterization and genomic and epigenomic analyses may enable the development of noninvasive, mechanism-informed, and personalized strategies for bladder cancer detection, surveillance, and risk stratification.

## Figures and Tables

**Table 1:** Serum metabolite concentrations in healthy controls and patients with NMIBC.

	Control (*n* = 52)	pTa (*n* = 25)	pT1 (*n* = 25)	*p*-Value
BMI (kg/m^2^)	23.65	25.78	24.22	0.144
	(21.55–28.19)	(23.40–29.32)	(21.97–28.76)	
Height (m)	1.70	1.68	1.70	0.672
	(1.63–1.75)	(1.62–1.76)	(1.65–1.80)	
Age (years)	71.50	66.00	71.00	0.095
	(66.00–76.00)	(64.00–70.00)	(66.00–79.00)	
Weight (kg)	73.50	72.00	75.00	0.695
	(65.00–80.00)	(64.50–86.50)	(65.00–82.50)	
Betaine (μM/L)	17.07	26.77 *	22.17	0.094
	(13.19–22.18)	(15.89–30.91)	(11.2–36.08)	
Carnitine (μM/L)	60.96	92.69 *	78.35 *	<0.0001
	(43.43–71.71)	(77.79–102.40)	(69.06–99.12)	
Choline (μM/L)	57.52	98.83 *	120.54 *	<0.0001
	(38.39–65.38)	(65.74–164.93)	(65.28–146.62)	
TMAO (μM/L)	0.85	1.95 *	1.82 *	<0.0001
	(0.80–1.45)	(1.42–2.00)	(1.33–2.71)	

Data are presented as median (interquartile range). Statistical significance was assessed using the Kruskal–Wallis test, followed by the Bonferroni-adjusted Mann–Whitney *U* test; * *p* < 0.05 vs. control. Abbreviations: pTa, noninvasive papillary urothelial carcinoma; pT1, lamina propria-invasive urothelial carcinoma; TMAO, trimethylamine-*N*-oxide; μM/L, micromoles per liter; BMI, body mass index.

**Table 2:** Diagnostic performance of individual serum metabolites and composite risk score in distinguishing NMIBC from healthy controls.

	AUC (95% CI)	Cutoff Value	Sensitivity	Specificity	Youden’s Index	*p*-Value
Betaine (µM/L)	0.659 (0.548–0.771)	23.44	0.540	0.846	0.386	<0.0001
Carnitine (µM/L)	0.836 (0.759–0.913)	66.07	0.840	0.712	0.552	<0.0001
Choline (µM/L)	0.802 (0.714–0.891)	67.34	0.760	0.788	0.548	<0.0001
TMAO (µM/L)	0.875 (0.812–0.939)	1.48	0.723	0.846	0.570	<0.0001
Risk score	0.958 (0.926–0.991)	12.86	0.90	0.89	0.785	<0.0001

The cutoff values were determined according to the maximum Youden index. Binary classification was performed based on these cutoffs, and intergroup comparisons were performed using Fisher’s exact test. Abbreviations: AUC, area under the curve; CI, confidence interval; TMAO, trimethylamine-*N*-oxide; µM/L, micromoles per liter.

**Table 3:** Contextual comparison of the reported diagnostic performance of selected urine-based tests and the serum metabolite panel identified in this study.

Test/Biomarker	Sensitivity	Specificity	Sample Type	Reference
Urine cytology	~0.48	~0.86	Urine	Babjuk et al., 2022 [6]
NMP22 (BladderChek^®^)	~0.56	~0.88	Urine	Wang et al., 2017 [30]
UroVysion^™^ FISH	~0.77	~0.98	Urine	Ke et al., 2022 [31]
Cxbladder	~0.77	~0.94	Urine (mRNA)	Oyaert et al., 2025 [8]
BTA stat^®^	~0.62	~0.52	Urine	Ecke et al., 2023 [32]
TMAO (µM/L)	0.723	0.846	Serum	Our study
Carnitine (µM/L)	0.840	0.712	Serum	Our study
Choline (µM/L)	0.760	0.788	Serum	Our study
Composite risk score	0.90	0.89	Serum	Our study

The reported sensitivity and specificity values for urine-based tests are derived from previous studies and may vary depending on the study design and patient population. A direct head-to-head comparison was not performed.

## Data Availability

The datasets generated and analyzed during the current study are not publicly available due to ethical restrictions related to patient confidentiality. De-identified data may be available from the corresponding author upon reasonable request and with permission from the relevant ethics committees.

## Supplementary Materials

The following supporting information can be downloaded at: https://www.mdpi.com/article/10.3390/ijms27083591/s1.

## Abbreviations

The following abbreviations are used in this manuscript:

AUC	Area under the curve
BC	Bladder cancer
BMI	Body mass index
BTA	Bladder tumor antigen
CI	Confidence interval
CT	Computed tomography
DNA	Deoxyribonucleic acid
ER	Endoplasmic reticulum
FISH	Fluorescence in situ hybridization
FGFR3	Fibroblast growth factor receptor 3
FMO3	Flavin-containing monooxygenase 3
GLOBOCAN	Global Cancer Observatory
IL-6	Interleukin-6
IQR	Interquartile range
LC–MS/MS	Liquid chromatography–tandem mass spectrometry
MRI	Magnetic resonance imaging
MRM	Multiple reaction monitoring
NMP22	Nuclear matrix protein 22
NF-κB	Nuclear factor kappa B
NMIBC	Non-muscle-invasive bladder cancer
OR	Odds ratio
pTa	Pathological Ta (non-muscle-invasive papillary tumor)
pT1	Pathological T1 (tumor invading subepithelial connective tissue)
pT2	Pathological T2 (tumor invading muscularis propria)
ROC	Receiver operating characteristic
ROS	Reactive oxygen species
SD	Standard deviation
SPSS	Statistical Package for the Social Sciences
TMA	Trimethylamine
TMAO	Trimethylamine-*N*-oxide
TNF-α	Tumor necrosis factor alpha
TERT	Telomerase reverse transcriptase
TUR-BT	Transurethral resection of bladder tumor
UHPLC	Ultra-high-performance liquid chromatography
USG	Ultrasound imaging
VEGF	Vascular endothelial growth factor
