# Supplementary material for: Serum Trimethylamine-N-Oxide and Its Precursors as a Diagnostic Biomarker Panel for Non-Muscle-Invasive Bladder Cancer
Source: Int J Mol Sci. 2026 Apr 17;27(8):3591. doi: 10.3390/ijms27083591 (PMC13115581; doi:10.3390/ijms27083591)
Supplement: Supplementary file 1 [file ijms-27-03591-s001.zip › ijms-4201438-supplementary.pdf]

Supporting Information

Table S1. Spearman's correlation coefficients between serum metabolites and clinical variables.

	**Carnitine *(µM/L)***	**Choline *(µM/L)***	***TMAO (µM/L)***	**Betaine *(µM/L)***	**Age *(years)***	***p-value***
Choline *(µM/L)*	0.446 (0.270–0.593)					<0.0001
TMAO (µM/L)	0.449 (0.273–0.596)					<0.0001
		0.295 (0.101–0.468)				0.0026
Betaine *(µM/L)*	0.373 (0.186–0.533)					0.0001
		0.287 (0.093–0.461)				0.0034
			0.177 (−0.024 to 0.367)			0.0748
Age *(years)*	0.011 (−0.190 to 0.210)					0.92
		−0.066 (−0.263 to 0.135)				0.51
			0.033 (−0.168 to 0.232)			0.74
				−0.165 (−0.353 to 0.036)		0.10
BMI (kg/m^2)	0.117 (−0.085 to 0.310)					0.24
		0.097 (−0.105 to 0.291)				0.33
			0.198 (−0.002 to 0.383)			0.05
				−0.157 (−0.346 to 0.045)		0.32
					0.141 (−0.061 to 0.332)	0.16

Values are presented as Spearman's correlation coefficient (rs) with 95% confidence intervals. Two-tailed *p*-values are shown.

Table S2. Multivariate logistic regression analysis of serum metabolites in distinguishing NMIBC from healthy controls.

Variable	***Estimate (β)***	***p-value***	***Odds Ratio (95% CI)***
Betaine *(µM/L)*	–0.014	0.712	0.986 (0.915–1.062)
Carnitine *(µM/L)*	0.07141	0.001	1.074 (1.027–1.1123)
Choline *(µM/L)*	0.02485	0.004	1.025 (1.008–1.043)
TMAO (µM/L)	4.115	0.000	61.23 (7.201–520.7)

The overall model demonstrated statistical significance in the likelihood-ratio test ($\chi^2(4) = 88.18$; $p < 0.0001$). Carnitine, choline, and TMAO were significant independent predictors, whereas betaine did not reach statistical significance. Abbreviations: CI, confidence interval; OR, odds ratio; TMAO, trimethylamine-N-oxide.
